# Painful Elastofibroma Dorsi: A Report of a Case and a Brief Review of the Literature

**DOI:** 10.1155/2013/794247

**Published:** 2013-01-14

**Authors:** Evangelos Falidas, Dimitrios Arvanitis, Georgios Anyfantakis, Angelos Pazidis, Zacharoula Koukouli, Dimosthenis Miltiadou, Anastasia Koronaiou

**Affiliations:** ^1^Department of Surgery, Florina General Hospital, Egnatias 9, 53100 Florina, Greece; ^2^Department of Pathology, University of Thessaly, Biopolis, 41110 Larissa, Greece; ^3^Department of Radiology, NIMTS General Hospital of Athens, Monis Petraki 10-12, 11521 Athens, Greece

## Abstract

Elastofibroma dorsi (ED) is an uncommon, slow-growing, benign, soft tissue tumor of unclear pathogenesis, typically located at the subscapular region of elderly people. It may be unilateral or bilateral. Though many patients are asymptomatic, ED can cause local deformity and symptoms such as periscapular pain or discomfort. Herein we report a case of a 65-year-old woman with painful ED. Clinical features, radiodiagnostic, intraoperative, and pathologic findings, and a brief review of the literature are performed.

## 1. Introduction

Elastofibroma dorsi (ED) is a benign, soft tissue tumor found in the subscapular and infrascapular region between the thoracic wall, serratus anterior, and latissimus dorsi muscle. It was first described in 1961 by Jarvi and Saxen [1]. The international experience since then consists mostly of small series and occasional case reports. This fact not only limits the unanimous characterization of the tumour regarding conservative, surgical, and postoperative followup, but also strengthens the misbelief that ED is an extremely rare condition. Go et al. in a recent review of the literature identified 330 cases of elastofibroma between 1980 and 2009 [2]. We herein report a case of a symptomatic, unilateral ED in a 65-year-old woman, and we briefly perform a PubMed research adding 161 cases of ED from 2009 up today in the already existing literature [3–26].

## 2. Case Presentation

**Figure 1:**
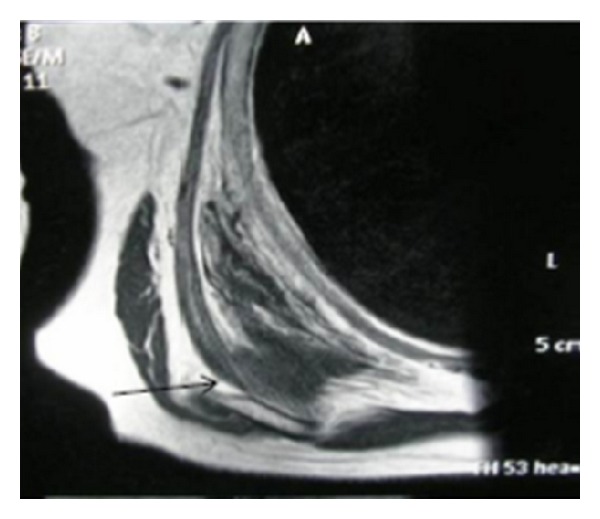
Axial MRI image indicating a hypointense rather round mass with signal intensity almost equal to surrounding muscles.

**Figure 2:**
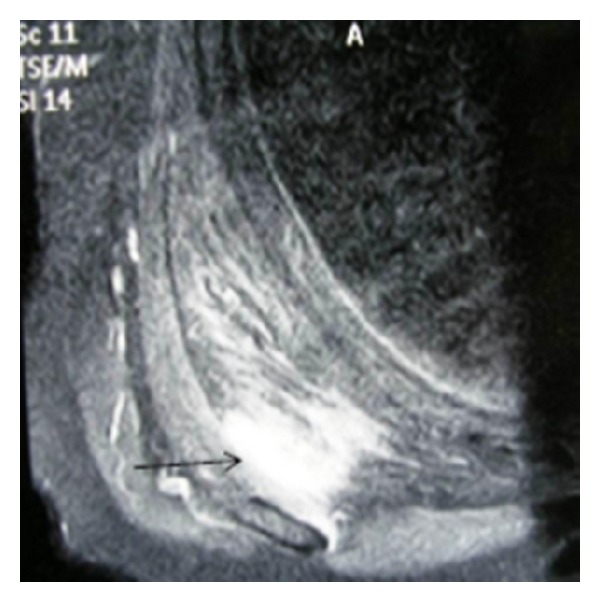
Fat suppression image after gadolinium demonstrating rather homogenous enhancement of the mass.

**Figure 3:**
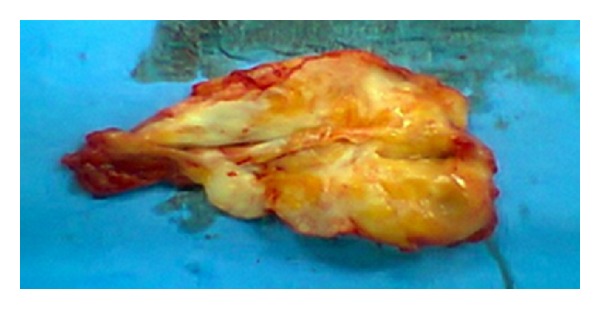
Surgical specimen of elastofibroma dorsi.

**Figure 4:**
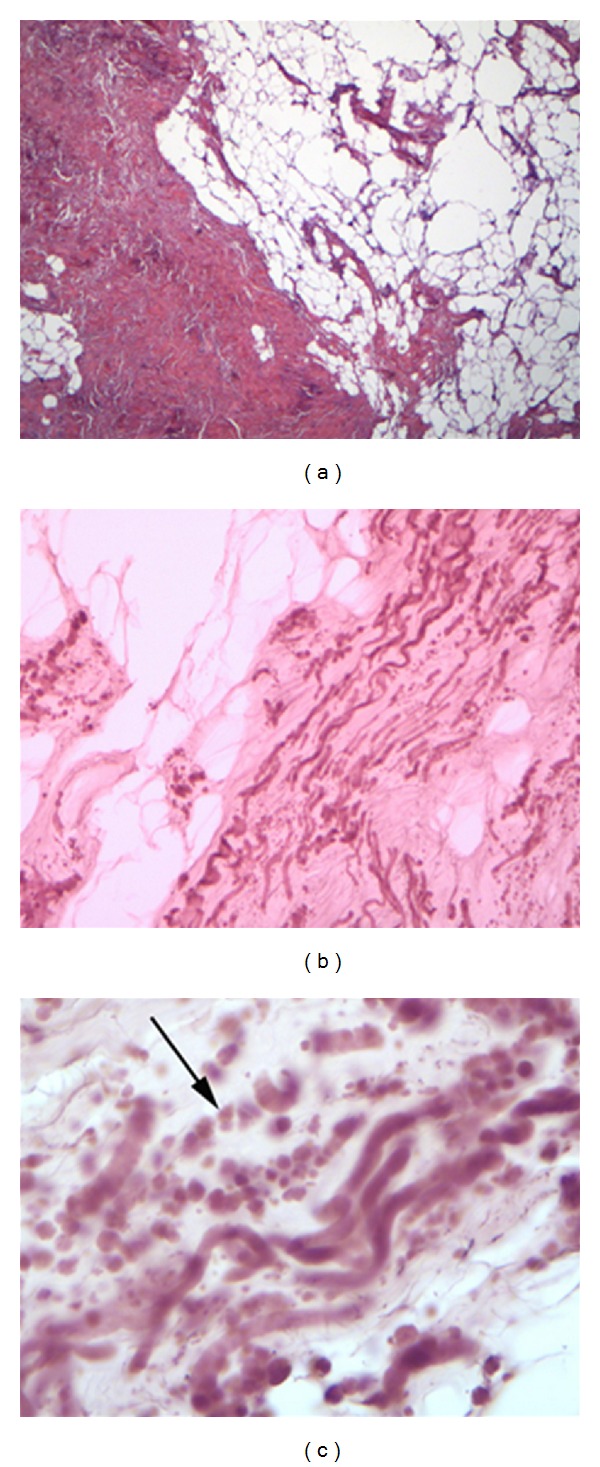
(a) A thick bundle of hypocellular collagenous tissue among fatty tissues. H/E stain. Magnification: ×25. (b) Shikata elastic tissue stain showing the configuration of serpentine elastic fibers. Magnification: ×100. (c) Shikata elastic tissue stain showing serpentine elastic fibers and broken elastic fibers arranged in a string of beads pattern (arrow). Magnification: ×400.

A 65-year-old woman with no significant medical history arrived at the outpatient facilities referring a painful shoulder mass, which had been slowly growing during the last five years. The mass was slightly visible in the neutral position. However, upon physical examination a firm and easily palpable mass at the inferior region of the right shoulder was found. Laboratory findings were within normal limits. She underwent magnetic resonance imaging (MRI) demonstrating a poorly circumscribed lesion with dimensions 67 × 29 × 45 mm located between the right lateral-posterior thoracic wall, the lower corner of the scapula, and serratus anterior muscle. The mass consisted of elements with signal intensity equal to muscles in T1 and T2, situated linearly and forming bundles. Linear areas with a signal intensity equal to fat were also dispersed among them. The lesion was nonhomogenously enhanced after contrast administration. No signs of malignancy were observed (Figures 1 and 2). With the patient lying in the prone position and with slightly abducted arm, local anesthesia was given. An incision was made over the palpable mass and the ill-defined tumor was removed (Figure 3). No intra- or postoperative complications occurred. Functional and sensational abnormalities of the operated arm were not observed. She was discharged 5 days after the initial observation. The pathology report described thick collagen bundles with entrapped islands of mature adipose tissue among them. The collagen bundles contained thick collagen fibres, few fibroblasts and a great number of elastic fibres, often fragmented into globules on a linear pattern one behind the other (Figures 4(a), 4(b), and 4(c)). The diagnosis of elastofibroma was posted. No signs of recurrence were found after 6 months of observation.

## 3. Discussion

Elastofibroma dorsi is a rare, benign, slow-growing soft tissue tumour with typical localization in the subscapular and infrascapular region, between the thoracic wall, serratus anterior, and latissimus dorsi muscle, often connected to the thoracic wall periosteum [1]. Unusual locations of ED such as orbit, mediastinum, and greater omentum have also been described [27].

Aetiology is unclear. It is strongly supported that ED is more common in people with high physical activity involving the shoulder [27, 28]. Thus, repetitive microtrauma due to friction between the scapula and the thoracic wall could be associated with the pathogenesis of ED leading to elastic degeneration of collagen or reactive hyperproliferation of fibroblastic tissue of the region [28, 29]. On the other hand, the reported incidence of ED in unusual sites not involved in mechanical overload could not justify the ED formation. Authors consider ED as the final outcome of a normal aging process [30] while others recognize a familial predisposition for ED formation [27]. 

Elastofibroma dorsi affects primarily the elderly, over 55 years of age, with a mean age of about 60 years at diagnosis [2]. However it is not impossible even for children to present with ED [27, 31]. Bilateral EDs seem to be quite common, up to 50% of the cases [2, 28]. ED unanimously is present more frequently in women rather than men (F : M ratio 3,9 : 1) [2]. The prevalence of the tumour in the elderly population varies from a low 2% as estimated with the use of CT [32] up to a high 24% for women and 11% for men in an autopsy series [28]. The conception that the ED is a very rare tumour seems to be unjustified and probably due to the benign nature, the small size, and the no causing symptoms of the tumor. Taking into consideration EDs as accidental findings in CT/MRI or during surgery for other reasons we have to assume that EDs are much more frequent.

When symptomatic, depending on the site and size of the lesion, ED may present as progressive swelling and discomfort or shoulder/back pain. Other symptoms include snapping of the scapula, stiffness of the shoulder, and restriction of shoulder movements. The physical examination may reveal a palpable, firm mass at the lower corner of the scapula, more prominent on abduction of the arm, usually immobile and probably due to its adherence to the surrounding tissue. Another mass may be present on the opposite shoulder, often smaller and clinically silent [2, 6, 21, 27, 33, 34].

Simple radiographs of the region cannot propose a diagnosis. The imaging modality of choice is MRI, which typically shows a well-defined and heterogeneous mass [2, 34]. The signal intensity is mostly low and similar in appearance to skeletal muscle with interspersed linear and curvilinear areas of higher signal intensity representing fat [31, 33, 35–37]. Administering a contrast agent, dishomogeneous enhancement may sometimes be observed demanding a biopsy for the differential diagnosis with malignant tumours [29, 31, 38]. In CT the heterogeneous structure of the mass is less clear, being less sensitive in visualizing the areas of adipose tissue [32, 37]. On PET-CT scan most of the tumours appear with mild or moderate diffuse metabolic activity [12, 14]; however hypermetabolic tumours have been described [14]. EDs present on ultrasound characteristic patterns; however, the examination is operator dependent and for that reason not always diagnostic [39].

Elderly patients or patients with bilateral tumours of typical localization and radiographic findings do not require biopsy, and the diagnosis of ED can be presumed. If the case is less typical an open biopsy or core needle biopsy must be performed. A fine needle biopsy is inadequate to get a representative tissue specimen. The differential diagnosis includes sarcoma, lipoma, fibroma, liposarcoma fibromuscular tumour, desmoid tumour, hemangioma, and hematoma, aggressive fibromatosis [2, 34]. Histological examination describes the characteristic patterns of the tumor. Altered elastic fibres are found in a collagenous matrix, mingled with fat cells that form islands of adipose tissue in various sizes [34]. Macroscopically the fibroelastic mass is not encapsulated and poorly defined, with a consistence that assimilates rubber. A “checkerboard pattern” characterizes the cut surface, created by strands of white and yellow tissues, that correspond to the fibroelastic and adipose component of the lesion [35]. Histologically, round-shaped collections of elastic fibres are found among fibrous, collagenous strands. These elastic fibres are eosinophilic, plump, elongated, and larger than regular ones, fragmented into disks or globules. As it may be difficult for these fibres to be visualised with hematoxyline-eosine-staining, especially during the frozen section procedure, an elastic stain (Elastic-van-Gieson) is needed to highlight them, staining them dark brown to black. The lesions are mostly hypocellular, containing fibrocystic and fibroblastic cells. A central dense core is common [2, 27, 31, 33–35]. Dense granular bodies may exist within the fibroblast cytoplasm, probably representing elastin or its precursors [34]. No atypia or mitotic activity is found. 

When the diagnosis of ED is definitive, its treatment depends on the symptoms it causes to the patient. Due to its benign nature, for the asymptomatic patient with ED no excision is needed, and clinical followup proves to be sufficient [2, 29]. In those cases in which the ED causes strain to the patient or the diagnosis is less clear, it should be excised. Wide or radical resection is unnecessary, and curative marginal resection is recommended [2, 34]. A postoperative seroma or hematoma [21, 34] sometimes occurs, easily treated through needle aspiration or evacuation if needed. Postoperative wound drainage can be used in advance. No postoperative neurologic abnormalities or muscle weakness of the shoulder or the arm has been reported. Recurrence is extremely rare and probably the result of incomplete excision [27, 31]. However, no case of malignant transformation has ever been described [2, 34].

## 4. Conclusion

Elastofibroma dorsi should be taken into consideration in the differential diagnosis of divers shoulder pathologies and seems to be more frequent than reported. It is strongly supported that MRI is imaging modality of choice to identify ED. Knowledge of this entity could avoid useless procedures specially in elderly and asymptomatic patients where a simple followup could be sufficient. In symptomatic patients, marginal resection is recommended.
